# Trends in traffic accident mortality and social inequalities in Ecuador from 2011 to 2022

**DOI:** 10.1186/s12889-024-19494-7

**Published:** 2024-07-21

**Authors:** Juan Pablo Holguín-Carvajal, Tamara Otzen, Antonio Sanhueza, Álvaro Castillo, Carlos Manterola, Georgina Muñoz, Fernanda García-Aguilera, Fernanda Salgado-Castillo

**Affiliations:** 1 Programa de Doctorado en Ciencias Médicas, Universidad de La Frontera, Temuco, Chile; 2 Facultad de Medicina, Universidad del Azuay, Cuenca, Ecuador; 3 Núcleo Milenio de Sociomedicina, Santiago, Chile; 4 Departamento de Especialidades Médicas, Universidad de La Frontera, Temuco, Chile; 5 Organización Panamericana de la Salud, Washington, USA; 6 Department of Public Health, Facultad de Medicina y Ciencia, Universidad San Sebastián, Concepción, Chile; 7 Millennium Nucleus for the Evaluation and Analysis of Drug Policies (nDP), Santiago, Chile; 8 Universidad Central del Ecuador, Quito, Ecuador

**Keywords:** Traffic accidents, Mortality, Trends, Ecuador

## Abstract

**Background:**

Traffic accidents (TA) remain a significant global public health concern, impacting low—and middle-income countries. This study aimed to describe the trend in TA mortality and inequalities in Ecuador for 2011–2022, distributed by year, gender, age group, geographical location, type of accident, and social inequalities.

**Methods:**

An ecological study was conducted using INEC national-level data on TA fatalities in Ecuador. Mortality rates were calculated per 100,000 population and analyzed by year, gender, age group, geographic region, and accident type. Annual percentage variation (APV) was determined using linear regression models. Inequality analyses examined associations between TA mortality and socioeconomic factors like per capita income and literacy rates. Complex measures such as the Slope Inequality Index (SII) were calculated to assess the magnitude of inequalities.

**Results:**

There were 38,355 TA fatalities in Ecuador from 2011 to 2022, with an overall mortality rate of 19.4 per 100,000 inhabitants. The rate showed a non-significant decreasing trend (APV − 0.4%, *p* = 0.280). Males had significantly higher mortality rates than females (31.99 vs. 7.19 per 100,000), with the gender gap widening over time (APV 0.85%, *p* = 0.003). The Amazon region had the highest rate (24.4 per 100,000), followed by the Coast (20.4 per 100,000). Adults aged ≥ 60 years had the highest mortality (31.0 per 100,000), followed by those aged 25–40 years (28.6 per 100,000). The ≥ 60 age group showed the most significant rate decrease over time (APV − 2.25%, *p* < 0.001). Pedestrians were the most affected group after excluding unspecified accidents, with a notable decreasing trend (APV − 5.68%, *p* < 0.001). Motorcyclist fatalities showed an increasing trend, ranking third in TA-related deaths. Lower literacy rates and per capita income were associated with higher TA mortality risks. Inequality in TA mortality between provinces with the highest and lowest per capita income increased by 247.7% from 2011 to 2019, as measured by the SII.

**Conclusion:**

While overall TA mortality slightly decreased in Ecuador, significant disparities persist across demographic groups and geographic regions. Older adults, males, pedestrians, and economically disadvantaged populations face disproportionately higher risks. The increasing trend in motorcycle-related fatalities and widening socioeconomic inequalities are particularly concerning.

## Introduction

Traffic accidents (TA) are a significant global health concern, encompassing incidents involving vehicles designed for transporting people, animals, or goods. The World Health Organization (WHO) has classified these events under “Chapter XX: External Causes of Morbidity and Mortality” in the International Statistical Classification of Diseases and Related Health Problems, 10th revision (ICD-10), using codes V01-V99 to generate comprehensive global statistics [1].

The importance of addressing TA is underscored by their inclusion in the United Nations Sustainable Development Goals (SDGs). Specifically, SDG 3, which focuses on Health and Well-being, aims to decrease the global number of deaths and injuries from TA by 2030 [2]. The WHO has highlighted the effectiveness of various road safety measures implemented worldwide to achieve this goal [3].

Worldwide data on TA reveals a profoundly concerning situation, highlighting this issue’s severity and widespread impact across different regions and populations. In 2019, WHO reported 1,282,150 TA-related deaths globally, with higher mortality rates among men and in low-income countries. The disparity in TA mortality rates across World Bank income groups labels them as low income (28.3/100,000 population), lower-middle-income (17.3/100,000), upper-middle income (16.8/100,000), and high income (8.4/100,000) [4].

These figures represent a significant loss of life and have substantial economic implications, with TA accounting for approximately 3% of the Gross Domestic Product (GDP) in most countries [5].

The gravity of this public health issue, recognized globally since 2004, is further emphasized by trend analyses projecting TA to become the fifth leading cause of death by 2030 [6, 7]. In the Americas, the impact of TA is particularly severe among younger populations. In 2012, TA was the primary cause of death for individuals aged 5–14 and the second leading cause for those aged 15–44, resulting in 149,252 fatalities. The Andean Region of South America reported a notably high TA mortality rate of 20.9/100,000 population [6].

The burden of TA varies across income levels, with data from 2019 showing TA mortality ranking seventh in low-income countries and tenth in lower-middle and upper-middle-income countries [8]. This variance underscores the need for targeted interventions tailored to different economic contexts.

Ecuador provides an interesting case study in TA trends. A study from 2000 to 2015 found an average TA mortality rate of 11.4/100,000 [9], which was lower than the Americas’ average of 15.8/100,000 [6]. However, a more comprehensive study, spanning 2000–2019, revealed a higher average mortality rate of 18.2/100,000 inhabitants. This study also identified a significant decrease in TA, with an annual percentage variation (APV) of -8.54% each year [10].

Ecuadorian recent data from 2020 have demonstrated a decrease in TA-related deaths, recording 16,972 TA resulting in 2,600 deaths (15.3%). This represents a 31% reduction from 2019, with a mortality rate of 14.8 per 100,000 population. Notably, 64% of these fatalities happened at the accident site, while the remaining 36% occurred in hospitals or care centers [11].

## State of the art

Traffic accidents in Ecuador represent a growing public health issue of growing importance, as evidenced by studies conducted over the past two decades. This analysis synthesizes the most relevant findings, offering a comprehensive perspective on the current situation, emerging trends, and challenges in road safety in the country.

### Evolution of TA and mortality patterns

The mortality rate increased in both female and male populations, at 2.05 and 3.29 per year, respectively. The authors conclude that, despite the decrease in incidents, their severity and lethality have considerably increased [10].

Another study identified a significant correlation between the TA mortality rate and motorization and injury rates, validating this fact. These findings suggest that the increase in the vehicle fleet and the greater severity of accidents directly contribute to the rise in road mortality [9].

### Gender and age disparities in TA

The analyzed studies have shown that there is a pronounced gender disparity in TA mortality; the male population presents a significantly higher risk of dying in TA, constituting approximately 80% of fatal victims; moreover, male drivers exhibit a higher incidence of risky driving behaviors and a greater likelihood of being involved in road accidents [10, 12].

Regarding the age dimension, the study’s authors [13] provide a perspective on the elderly population’s vulnerability to TA in Ecuador. Although this demographic segment registers a lower incidence of injuries compared to younger age groups, it presents higher mortality rates, suggesting a greater susceptibility to fatal injuries due to physical frailty [13].

### Etiology and precipitating factors of TA

It has been identified that distracted driving is the primary causal factor of road accidents, with a prevalence of 56.8%. Other significant factors include driving under the influence of alcohol and speeding [14].

In a recent study, these factors were emphasized, where specific high-risk behaviors among drivers were broadly identified in [12]:

- Disregard for speed limits, particularly during nighttime or early morning hours.
- Non-compliance with traffic light signaling at intersections.
- Manifestations of aggressive driving, such as expressing anger through gestures.
- Judgment errors, such as underestimating vehicle speed in overtaking maneuvers.

Additionally, this study highlights that taxi drivers, followed by bus and truck operators, exhibit the highest scores in three of the four main dimensions of the Spanish Driving Behavior Questionnaire (SDBQ): violations, lapses, and aggressive behaviors. This suggests a greater propensity for unsafe driving behaviors and, consequently, involvement in road accidents [12]; it also includes a new dimension, which considers driving hours a significant risk factor.

### Social and geographical inequities in TA

It is necessary to underline the existence of significant disparities in TA mortality based on socioeconomic and geographical factors [9]; low-income countries, such as Ecuador, present higher TA fatality rates. Drivers with longer daily exposure are more susceptible to unsafe behaviors, which could partially explain geographical disparities in accident rates [12].

This research emphasizes the critical importance of continued investigation into TA and associated mortality globally, particularly in Ecuador. This literature review reveals significant knowledge gaps despite TA’s substantial public health burden. The study aims to comprehensively analyze TA impact in Ecuador, focusing on epidemiological trends and disparities. The objective of exploring mortality patterns across various parameters is to gather evidence-based prevention strategies and policy improvements. Future research should prioritize road safety enhancements, emergency response optimization, and targeted interventions to reduce TA-related morbidity and mortality, ultimately promoting safer transportation environments.

## Methods

### Aim

To describe the trend in TA mortality and inequalities in Ecuador for 2011–2022, distributed by year, gender, age group, geographical location, type of accident, and social inequalities.

### Design

An ecological study was conducted using aggregated national-level data on traffic accidents in Ecuador from 2011 to 2022.

### Subjects

The population of interest consisted of all individuals who died due to TA in Ecuador between 2011 and 2022, obtained from the databases of the National Institute of Statistics and Census (INEC) of the Republic of Ecuador [15]. The general data on the population and live births were obtained from the same database, based on the national population projection distributed by age groups and provinces, as well as data on the number of vehicles registered at the national level and by province [16].

### Study variables

The variables were year (2011 to 2022); geographic region (Coast region: El Oro, Esmeraldas, Guayas, Los Ríos, Manabí, Santo Domingo, Santa Elena; Sierra: Azuay, Bolivar, Cañar, Carchi, Cotopaxi, Chimborazo, Imbabura, Loja, Pichincha, Tungurahua; Amazon: Morona Santiago, Napo, Pastaza, Zamora Chinchipe, Sucumbíos, Orellana; Insular: Galápagos; and Undelimited area); gender (male and female); age group (< 16 years; 17–24 years; 25–40 years; 41–59 years; >60 years); and accident type coded according to ICD-10 [1].

### Data analysis

Exploratory analyses were conducted using descriptive statistics for percentages, central tendency, and variability. Traffic accident mortality rates (TAMR) were calculated with the number of deaths as the numerator and population as the denominator per 100,000 inhabitants. Absolut risk (AR) was determined by geographic distribution, sex, age group, and accident type. Annual percentage variation (APV) in rates was analyzed using linear regression models with 95% confidence intervals and p-values [17]; for this, the natural logarithm (ln) of the ratios was calculated, and then a linear regression analysis was performed in IBM^®^ SPSS^®^ Statistics version 27 (the dependent variable was the ln and the independent variable was each year in study), obtaining as a result the βone and the error of β1 to calculate the APV in Microsoft^®^ Excel for Mac version 16.57 using the statistical formula: *APC* = { e*xp*(β1) − 1 } x 100, finally, the confidence intervals were calculated using the statistical formula: CI = { e*xp*(β1 ± (β1error x 1,96)) − 1 } x 100. Additionally, TAMR per 10,000 vehicles was calculated.

Further analyses included examining inequalities in traffic accident mortality as a sole health indicator, annual per capita income (PCI) and literacy rates as socioeconomic stratifiers, and live births per province as the demographic stratifier. Simple measures of absolute gaps (AG) and relative gaps (RG), as well as complex measures like the Slope Inequality Index (SII), were calculated using simple linear regression models [18].

The inequalities analyses were performed using EquiGap^®^ macro for Microsoft^®^ Excel developed by the EWEC-LAC metrics and monitoring working group [18].

## Results

### General mortality

During the study period, 38,355 individuals died due to traffic accidents in Ecuador, of whom 31,187 (81.3%) were men. The TAMR in this period was 19.4 per 100,000 inhabitants (Table 1).

The TAMR between 2011 and 2022 exhibited a decreasing APV of -0.4% (95% CI= -1.15; 0.31; *p* = 0.280), which was not statistically significant (Table 1).

From 2011 to 2016, 19,123 people died due to TA. The TARM was 20.08/100,000 population, revealing a decreasing statistically significant APV of -1.22% (95% CI= -2.03; -0.40; *p* = 0.043). In the period 2017 to 2022, 19,232 people died by TA (18.79 per 100,000 inhabitants). The APV was increasing 1.01% (95% CI= -1.79; 3.88; *p* = 0.523), not being significant.

The years with the highest TAMR were 2011 (22.0 per 100,000 inhabitants) and 2022 (21.7 per 100,000 inhabitants), whereas the years with the lowest TAMR were 2020 (14.9 per 100,000 inhabitants) and 2016 (18.0 per 100,000 inhabitants) (Table 2).

### Mortality by geographic region

**Fig. 1:**
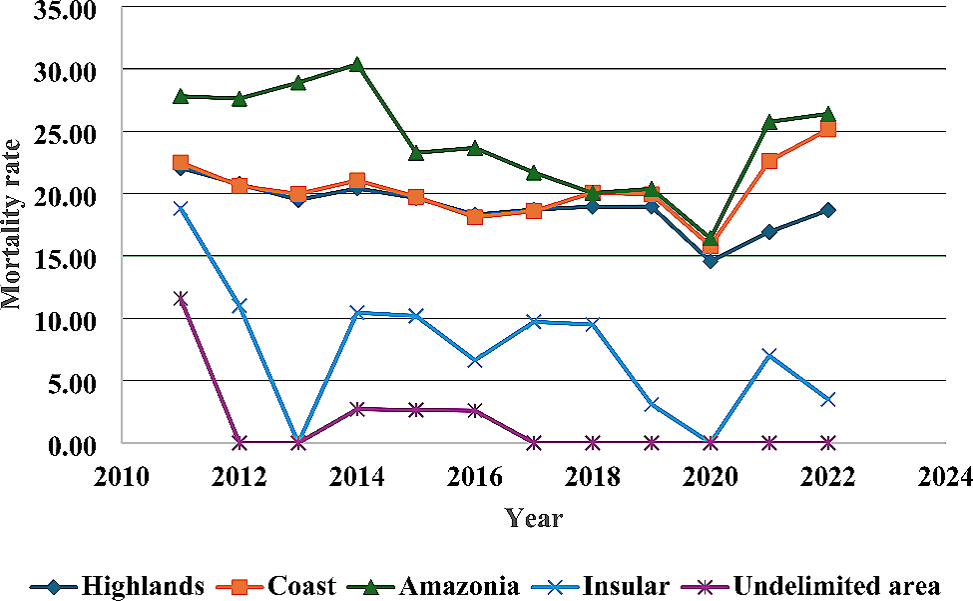
Traffic accident mortality rate per 100,000 inhabitants by region. Source: INEC (National Institute of Statistics and Census of Ecuador). Performed by: Authors

According to geographic region, the highest rates in the period were in the Amazon and Coast regions, with 24.4 per 100,000 inhabitants and 20.4 per 100,000 inhabitants, respectively (Table 1; Fig. 1).

Regarding provinces, the highest TAMR was registered in Santo Domingo (30.6 per 100,000 inhabitants) and Sucumbíos (29.6 per 100,000 inhabitants), while the lowest TAMR was for the undelimited area (1.9 per 100,000 inhabitants) and Galápagos (7.9 per 100,000 inhabitants) (Table 1).

The trend analysis among geographic regions revealed that the undelimited area and the insular region had the highest APV (-5.3%; 95% CI: -9.56 to -0.82; *p* = 0.044; and − 4.2%; 95% CI: -10.29 to 2.24; *p* = 0.224, respectively) (Table 1).

**Table 1:** Traffic accident mortality rate per 100,000 inhabitants from 2011 to 2022 by gender, region, provinces, age range, and male vs. female rate ratio with respective 95% CI and *p*-value

		TAMR	APV	95% CI		*p*
	2011–2016	20.08	-1.22	-2.03	-0.40	0.043
	2017–2022	18.79	1.01	-1.79	3.88	0.523
	**General rate** **(2011–2022)**	**19.43**	**-0.42**	**-1.15**	**0.31**	**0.280**
Gender	Male	31.99	-0.24	-1.00	0.52	0.545
	Female	7.19	-1.11	-1.87	-0.35	0.018
Region	Sierra	18.96	-0.93	-1.46	-0.41	0.006
	Coast	20.35	0.02	-0.85	0.90	0.966
	Amazon	24.36	-1.17	-2.29	-0.04	0.070
	Insular	7.49	-4.23	-10.29	2.24	0.224
	Undelimited area	1.63	-5.29	-9.56	-0.82	0.044
Provinces	Azuay	14.15	0.78	-0.92	2.50	0.394
	Bolívar	20.64	0.81	-0.79	2.44	0.344
	Cañar	23.22	0.62	-0.62	1.87	0.353
	Carchi	19.71	-0.30	-1.56	0.97	0.653
	Cotopaxi	27.72	-0.95	-2.32	0.43	0.207
	Chimborazo	24.76	-0.45	-1.42	0.54	0.397
	El Oro	22.16	-0.53	-1.63	0.58	0.369
	Esmeraldas	15.69	-0.06	-1.82	1.74	0.949
	Guayas	19.24	-0.54	-1.33	0.25	0.210
	Imbabura	17.94	-2.67	-3.99	-1.35	< 0.01
	Loja	12.65	-0.68	-2.38	1.04	0.455
	Los Ríos	27.31	0.51	-0.39	1.42	0.290
	Manabí	15.65	0.16	-0.60	0.94	0.686
	Morona Santiago	23.84	0.88	-1.17	2.97	0.421
	Napo	24.15	-1.78	-3.41	-0.12	0.062
	Pastaza	17.95	-0.02	-2.70	2.73	0.988
	Pichincha	17.83	-0.82	-1.41	-0.24	0.021
	Tungurahua	18.68	-0.93	-2.00	0.14	0.120
	Zamora Chinchipe	17.10	-1.46	-3.22	0.34	0.142
	Galápagos	7.49	-4.23	-10.29	2.24	0.224
	Sucumbíos	29.62	-1.93	-3.76	-0.06	0.070
	Orellana	27.56	-1.71	-3.24	-0.15	0.058
	Santo Domingo	30.63	-0.16	-0.80	0.48	0.634
	Santa Elena	13.07	0.59	-0.77	1.97	0.418
Age Range	0 to 16 years	5.10	-2.05	-3.23	-0.86	0.007
	17 to 24 years	26.84	-1.02	-1.52	-0.53	< 0.01
	25 to 40 years	28.57	0.52	-0.45	1.50	0.316
	41 to 59 years	21.40	-1.26	-1.87	-0.65	< 0.01
	60 or more years	31.05	-2.25	-3.07	-1.43	< 0.001

^1^*TAMR* Traffic accident mortality rate, ^2^*APV* Annual percentage variation, ^3^*CI* Confidence interval, ^4^*p*: *p*-value

Source: *INEC* (National Institute of Statistics and Census of Ecuador)

Performed by: Authors

**Table 2:** Traffic accident mortality rate per 100,000 inhabitants from 2011 to 2022 by year, gender, and male vs. female rate ratio

Year	*n*	TAMR	Male	Female	M/F AR
2011	3368	22.06	36.35	8.01	4.54
2012	3186	20.53	32.88	8.39	3.92
2013	3109	19.71	31.95	7.69	4.15
2014	3323	20.73	33.50	8.20	4.09
2015	3157	19.39	31.85	7.17	4.44
2016	2980	18.03	29.46	6.82	4.32
2017	3079	18.35	29.72	7.20	4.13
2018	3244	19.06	31.22	7.13	4.38
2019	3279	18.99	31.30	6.93	4.52
2020	2600	14.85	24.96	4.94	5.05
2021	3345	19.75	33.67	6.52	5.16
2022	3685	21.75	37.01	7.26	5.10
2011–2022	38,355	19.39	31.99	7.19	4.48

^1^n: number of deaths, ^2^TAMR Traffic accident mortality rate, ^3^ M/F Male/Female, 4AR Absolut risk

Source: INEC (National Institute of Statistics and Census of Ecuador)

Performed by: Authors

There was a 1.2 times higher risk of mortality due to TA in the Amazon compared to the Coast, with a decreasing APV of -1.2% (95% CI: -1.83 to -0.56; *p* = 0.004). It was evident that the most significant difference in rates between the provinces of Santo Domingo de los Tsáchilas and Galapagos (AR = 3,7) represented an APV with an annual increase of 4.3% in the rates (95% CI: -0.31 to 9.21; *p* = 0.097) (Table 3).

**Table 3:** The absolute risk of TAMR and the annual percentage variation of absolute risk with 95% CI in Ecuador from 2011 to 2022

	AR	APV	95% CI		*p*
Male/Female	4.48	0.85	0.41	1.29	0.003
Coast/Sierra	1.08	0.98	0.43	1.53	0.005
Amazon/Coast	1.20	-1.20	-1.83	-0.56	0.004
Santo Domingo/Galápagos	3.68	4.34	-0.31	9.21	0.097
Pedestrian/Bus	35.57	-7.71	-12.63	-2.51	0.016
Unspecified Transport/Pedestrian	4.18	6.93	6.01	7.86	< 0.001
60 and over/0 to 16 years	6.18	-0.19	-1.40	1.05	0.771

^1^AR Absolute risk, ^2^APV Annual percentage variation, ^3^CI: Confidence interval, ^4^p p-value

Source: INEC (National Institute of Statistics and Census of Ecuador)

Performed by: Authors

### Mortality by gender

**Fig. 2:**
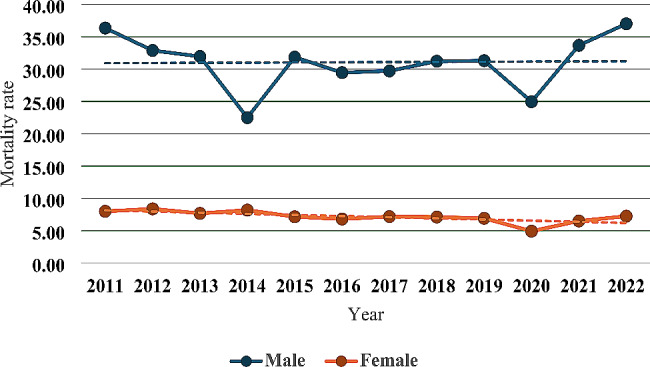
Traffic accident mortality rate per 100,000 inhabitants by gender. Source: INEC (National Institute of Statistics and Census of Ecuador). Performed by: Authors

Regarding gender, it was identified that men have a higher TAMR than women every year, with the highest TAMR for men in 2022 being 37.0 per 100,000 inhabitants, while for women, it was 8.4 per 100,000 inhabitants in 2012. The lowest TAMR in men and women was in 2020, with 24.9 per 100,000 inhabitants and 4.9 per 100,000 inhabitants, respectively (Table 2; Fig. 2).

Additionally, in 2021, the TAMR was 5.2 times higher in men than women, while in 2012, it was 3.9 times higher in men than women. The average male-to-female AR for the 12 years of the study was 4.5 (Table 3).

During the study period, women showed a higher APV in TAMR, at -1.11% decrease (CI=-1.87 to -0.35; *p* = 0.018) (Tables 1 and 2).

For the period 2011–2022, it was confirmed that the absolute risk of TA mortality in men compared to women was 4.5 times higher (AR = 4.5), indicating an annual increase associated with an APV of 0.9% (CI = 0.41–1.29; *p* < 0.01) (Tables 1 and 3).

### Mortality by age group

Analyzing the TAMR according to age groups, the highest mortality was observed in the ≥ 60 years group (31.0 per 100,000 inhabitants) and the 25 to 40 years group (28.6 per 100,000.

inhabitants), while the lowest rate was in the 0 to 16 years group (5.1 per 100,000 inhabitants). This trend remained consistent throughout the study period except for 2020 when the ≥ 60 years group exhibited the lowest rate (19.9 per 100,000 inhabitants) (Table 1).

The overall average age of fatalities due to TA was 37.8 years (Standard Deviation (SD) = 20.1). For men, it was 37.3 (SD = 19.0), and for women, 40.0 (SD = 24.1). The year with the lowest average age was 2022 (36.9 years, SD = 17.9), and the highest was 2017 (38.7 years). The differences for each year remained constant (APV = 0.03%; *p* = 0.718; 95% CI: -0.12 to 0.17) (Table 4).

**Table 4:** Averages of age fatalities due to traffic accidents in Ecuador from 2011 to 2022

Year	General				Male				Female				
	x	SD	Min	Max	x	SD	Min	Max	x	SD	Min	Max	Diff
2011	37.11	20.86	1	99	36.94	19.86	1	99	37.86	24.82	1	99	0.9
2012	37.03	20.58	1	99	36.73	19.59	1	99	38.17	24.00	1	99	1.4
2013	37.33	20.63	1	103	36.63	19.43	1	99	40.19	24.72	1	103	3.6
2014	37.91	20.58	1	100	37.5	19.54	1	100	39.55	24.28	1	99	2.1
2015	37.75	20.79	1	101	36.76	19.30	1	101	42.08	25.91	1	93	5.3
2016	38.83	20.80	1	98	38.37	19.61	1	95	40.79	25.14	1	98	2.4
2017	38.74	20.89	1	102	38.23	19.55	1	98	40.81	25.51	1	102	2.6
2018	38.65	20.70	1	101	37.98	19.73	1	100	41.56	24.22	1	101	3.6
2019	38.68	20.44	1	102	38.06	19.54	1	99	41.43	23.85	1	102	3.4
2020	37.16	18.69	1	109	36.85	17.92	1	109	38.71	22.09	1	99	1.9
2021	37.17	18.46	1	110	36.73	17.43	1	110	39.31	22.77	1	96	2.6
2022	36.99	17.97	1	99	36.3	16.88	1	99	40.34	22.22	1	98	4
2011- 2022	37.78	20.12	1	101.9	37.26	19.04	1	100.7	40.07	24.13	1	99.08	2.82

^1^x: Average age, ^2^*SD* Standard deviation, ^2^*Min* Minimum age, ^3^*Max* Maximun age, ^4^*Diff* Average age difference

Source: INEC (National Institute of Statistics and Census of Ecuador)

Performed by: Authors

Regarding the rate ratio in the ≥ 60 years group, a value of 6.4 times higher TAMR compared to the 0 to 16 years group was identified, with an annual decrease of -0.19% (95% CI: -1.40; 1.05; *p* = 0.771) (Table 3).

### Mortality by type of accident

Considering the TAMR, it was reported that between 2011 and 2022, there were 6,698 (17.5%) fatalities due to “pedestrian injured in transport accidents (ICD-10 V01-V09)” and 22,121 (57.7%) fatalities due to “other unspecified transport accidents (ICD-10 V089)” (Table 5).

These causes remained constant as the most frequent throughout each year; from 2018 onwards, there was an increase in fatalities among “Motorcyclists or occupants of motorized 3-wheeled vehicles (ICD-10 V20-V39)” as follows: 2018 (15.0%), 2019 (15.3%), 2020 (13.2%), 2021 (16.8%), 2022 (13.2%), and a decrease in fatalities among “pedestrians injured in transport accidents (ICD-10 V01-V09)” as follows: 2018 (14.6%), 2019 (13.7%), 2020 (11.2%), 2021 (9.3%), 2022 (7.0%).

Analyzing the types of annual TA deaths between 2011 and 2022, mortality among “pedestrians” showed the most significant variation with a tendency to decrease (APV= -5.7%; 95% CI: -6.45 to -4.91; *p* < 0.001) (Table 5).

**Table 5:** Types of traffic accidents in Ecuador from 2011 to 2022, with their respective percentages and annual percentage rates variation of 95% CI

Types of traffic accidents	*N*	%	APV	CI 95%		*p*
Pedestrian	6698	17.46	-5.68	-6.45	-4.91	< 0.001
Cyclist	416	1.08	0.89	-1.60	3.44	0.503
Motorcyclist	5430	14.16	1.91	-0.41	4.29	0.138
Vehicle Occupant	998	2.60	-4.63	-7.20	-1.98	0.006
Van Occupant	485	1.26	2.40	-2.14	7.15	0.329
Heavy Vehicle Occupant	289	0.75	3.21	-1.02	7.62	0.169
Bus Occupant	387	1.01	2.25	-3.40	8.23	0.460
Other Transport	1133	2.95	-1.00	-7.26	5.69	0.770
Unspecified	22,121	57.67	0.84	0.03	1.67	0.070
Maritime, Aerial, Space	398	1.04	-4.54	-10.20	1.47	0.166

^1^*n* number of death, ^2^%: Percentage of fatalities, ^2^*APV*: Annual percentage variation, ^3^*I* Confidence interval, ^4^*p p*-value

Source: INEC (National Institute of Statistics and Census of Ecuador)

Performed by: Authors

Regarding the differences in mortality risk by type of TA, it was evident that “Other unspecified transport” had 4.2 times more TAMR than that occurring in “pedestrians,” with an APV of 6.9 (95% CI: 6.01 to 7.86; *p* < 0.001); while the highest risk of mortality from traffic accident was among “pedestrians,” 35.6 times more than “bus occupants,” with an annual decrease of -7.7% (95% CI: -12.63 to -2.51; *p* = 0.016).

A depth analysis was conducted, focusing on the vulnerable population ≥ 60 years group and the specific accident type “pedestrian” (ICD-10 V01-V09). This subgroup analysis revealed a total of 2204 fatalities, accounting for 5.74% of all TA-related deaths in the study period. The TAMR for this subgroup was calculated at 10.60/100,000 from 2011 to 2022. The trends analysis demonstrated a statistically significant decrease in mortality, with an APV of -5.67% (95% CI: -6.67 to -4.65; p < 0.001).

Additionally, the mortality rate per 10,000 vehicles from 2011 to 2022 was calculated, resulting in 15.8 per 10,000 vehicles. It was also observed that the year with the highest mortality was 2011 (22.6 per 10,000 vehicles), and the lowest rate was in 2020 (11.0 per 10,000 vehicles). The APV of TA mortality per 10,000 registered vehicles for the entire period decreased by -2.4% (95% CI: -2.98 to -1.75; *p* < 0.001).

### Inequality analysis

**Fig. 3:**
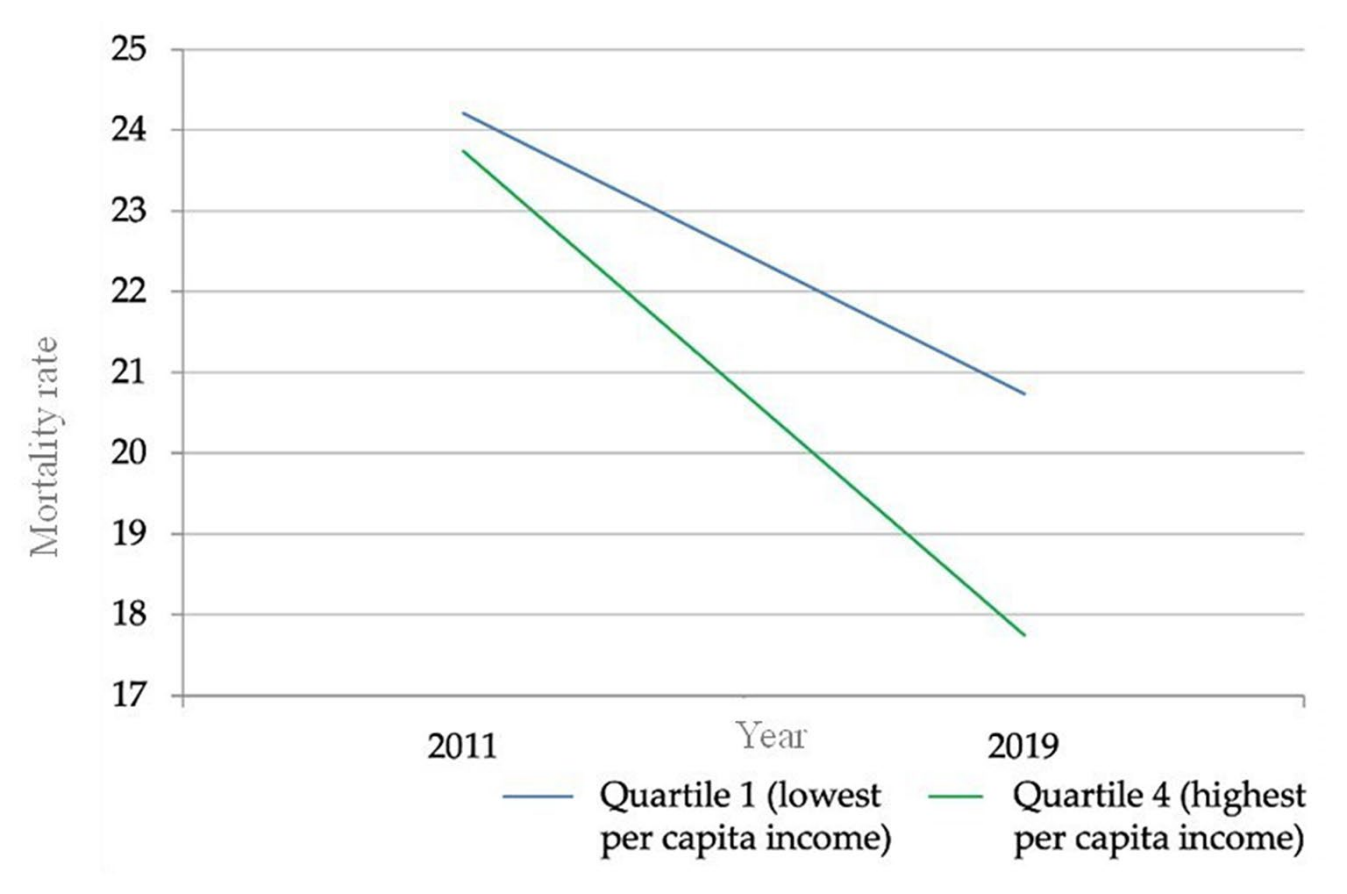
Change in the absolute gap in traffic accident mortality between 2011 and 2019. Source: INEC (National Institute of Statistics and Census of Ecuador). Performed by: Authors

It was identified that in 2011, there were 0.4 more deaths (AG) per 100,000 live births due to TA in the provinces with the lowest PCI compared to the provinces with the highest PCI; whereas, in 2019, there were 2.9 more deaths (AG) per 100,000 live births due to traffic accidents in the provinces with the lowest PCI compared to those with the highest PCI, representing a 500% increase in the AG between 2011 and 2019 (Fig. 3).

In 2011, the risk of mortality due to TA in the group of provinces with the lowest PCI was 1.0 times higher (RG) than for the group of provinces with the highest PCI, while in 2019, the risk of mortality due to TA in the group of provinces with the lowest PCI was 1.1 times higher (RG) than in the group of provinces with the highest PCI, indicating a 14.5% point increase in the AR between 2011 and 2019 (Fig. 3).

Calculating the SII in TA mortality in provinces stratified by PCI found that inequality increased by 247.7% between 2011 and 2019.

The analysis of the TAMR per 100,000 live births (mortality rates) in the years 2011 and 2019, when compared with the equity stratifier (PCI) and categorized by quintiles (Q1 to Q4, ranging from least advantageous condition to most advantageous condition), reveals that the highest mortality rate is predominantly observed in Q1 and the lowest in Q4 for both years. Concerning simple metrics, it is noted that the equity stratifier (PCI) registered a value of 0.48 (95% CI: -17.01 to 17.96) in BA in 2011 and 2.98 (95% CI: -14.57 to 20.53) in 2019, along with a value of 1.02 (95% CI: 0.49 to 2.12) in BR in 2011 and 1.17 (95% CI: 0.47 to 2.92) in 2019. These figures indicate the most significant departure from the equity condition, reflecting the greatest degree of inequality concentrated among populations with the most and slightest social advantage, respectively (Fig. 3).

**Fig. 4:**
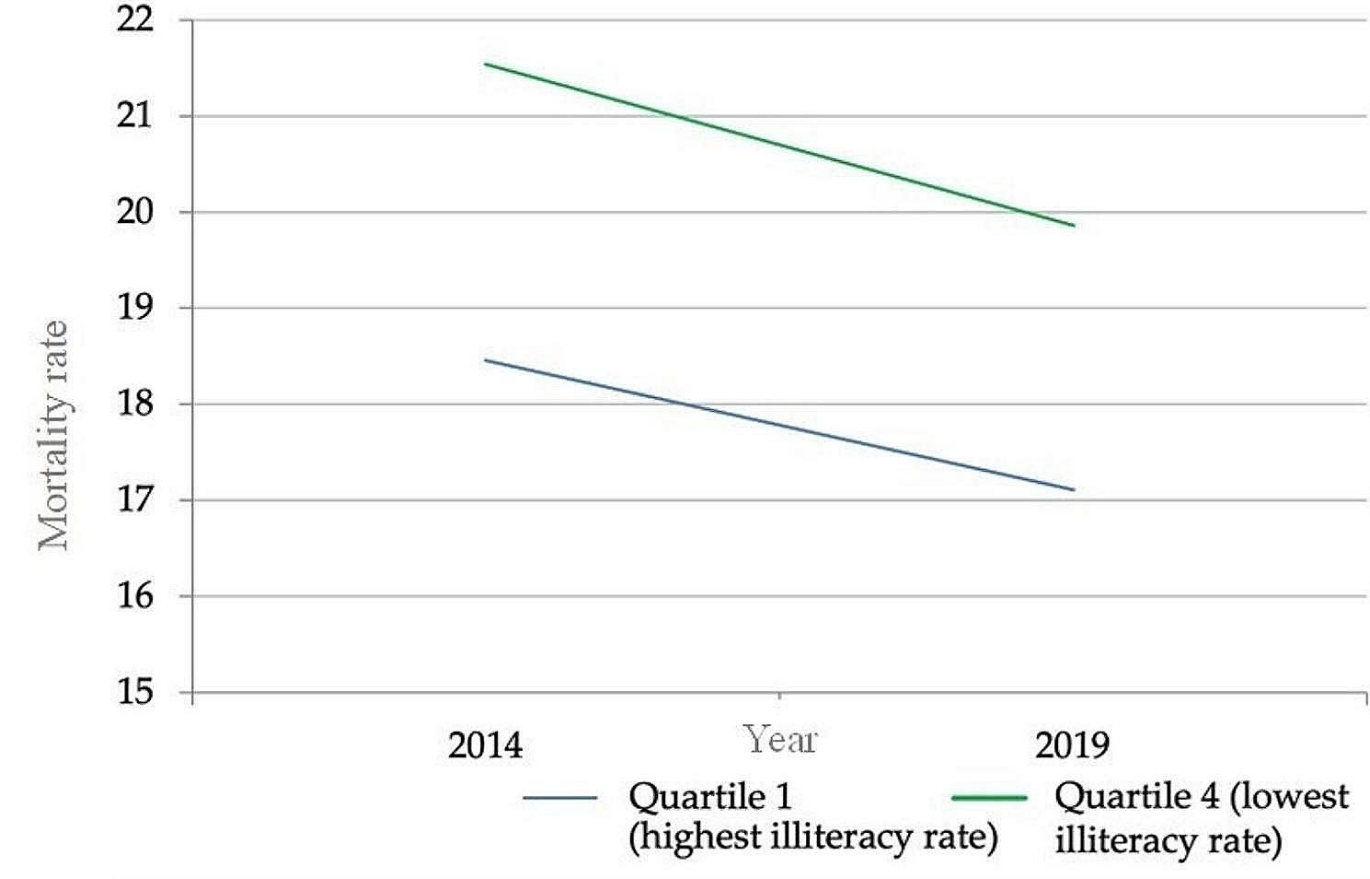
Change in the Absolute Gap in Traffic Accident Mortality between 2014 and 2019. Source: INEC (National Institute of Statistics and Census of Ecuador). Performed by: Authors

In 2014, there were 3.0 more deaths (AG) per 100,000 live births due to TA in the group of provinces with lower literacy levels compared to the group with higher literacy levels, whereas, in 2019, there were 2.7 more deaths (AG) per 100,000 live births due to TA in the group of provinces with lower literacy levels compared to those with higher literacy levels, signifying a 10.5% decrease in the AG between 2014 and 2019 (Fig. 4).

The risk of mortality due to TA in 2014 in the group of provinces with lower literacy levels was 1.1 times higher (RG) than in the group of provinces with higher literacy levels, a value very similar to 2019 (1.1 times higher (RG)), representing a 0.5% decrease in the RG (Fig. 4).

The analysis of the TAMR per 100,000 live births (mortality rates) in the years 2014 and 2019, when compared with the equity stratifier (illiteracy rate percentage) and categorized by quintiles (Q1 to Q4, ranging from least advantageous condition to most advantageous condition), indicates that the highest mortality rate is concentrated in Q1, while the lowest is in Q4 for both years. In terms of simple metrics, the equity stratifier (illiteracy rate percentage) demonstrated a value of -3.08 (95% CI: -18.3 to 12.14) in BA in 2014 and − 2.76 (95% CI: -17.47 to 11.95) in 2019; coupled with a value of 0.861 (95% CI: 0.4 to 1.84) in BR in 2014 and 0.86 (95% CI: 0.39 to 1.91) in 2019. These values represent the most significant deviation from the equity condition, reflecting the highest degree of inequality concentrated among populations with the most and slightest social advantage, respectively (Fig. 4).

When considering the SII in TA mortality in provinces stratified by the illiteracy rate percentage, it was found that inequality decreased by 18.0%.

Regarding complex metrics, the IPC stratifiers, with values of 247.7 in IDP, and the illiteracy rate, with values of 18.0 in IDP, describe the values furthest from the conditions of equity. Inequality is focused on populations with the most and slightest social advantage, respectively, over the years.

## Discussion

### Mortality by geographic region

Our study revealed significant variations in TA mortality rates across Ecuador’s provinces, aligning with findings from other Latin American countries. This geographic disparity suggests that regional factors such as road infrastructure, highway presence, lack of safety systems, traffic law enforcement, and emergency medical services play crucial roles in mortality outcomes [19–30]. The modest overall decrease of 0.42% in mortality rates, particularly notable in the Sierra region, contrasts with trends in countries like Brazil, Paraguay, Pakistan, Mongolia, and North Korea, where rates remain stable or are increasing [31, 32]. This underscores the importance of maintaining and enhancing road safety policies as a public health priority, as mandated by Ecuador’s law on terrestrial transport, transit, and road safety [33].

Our TAMR of 19.43 per 100,000 population from 2011 to 2022 is slightly higher than the average of 17.1 found in a study of 366 Latin American cities between 2011 and 2016 [34]. This comparison highlights the persistent challenge of traffic-related fatalities in Ecuador within the broader regional context. It emphasizes the need for continued efforts to improve road safety measures nationwide.

### Mortality by gender

Consistent with global and Latin American studies, our research found that men exhibit significantly higher TA mortality rates compared to women, with rates 3 to 6 times higher [19–24, 27–29, 31, 32]. This gender disparity may be attributed to differences in risk-taking behaviors, exposure to traffic, and occupational factors. The persistent nature of this disparity across various studies underscores the need for targeted interventions and policies addressing the specific risks male road users face.

A significant decrease in TAMR was observed during 2020 compared to 2019, with notable gender differences. The reduction was approximately 6 points for males and 2 points for females, likely due to COVID-19 lockdown measures [35, 36]. The more pronounced decrease in male mortality rates suggests that men may have been more affected by movement restrictions, possibly due to changes in work-related travel or leisure activities.

Interestingly, some studies present a contrasting perspective, revealing an increase in the case fatality rate of traffic incidents during the pandemic, particularly in developing countries [35, 37]. This paradoxical trend can be attributed to the initial collapse of healthcare systems due to COVID-19 pressures, highlighting the complex interplay between public health crises, healthcare system capacity, and traffic safety outcomes. This finding emphasizes the need for resilient healthcare systems that can maintain adequate trauma care even during times of crisis.

### Mortality by age group

The age group most impacted by TA mortality in our study was individuals over 60 years old, aligning with research from Colombia, where patients in this age range faced double the mortality risk from traffic accidents compared to younger patients [25]. However, another Colombian study using 2019 data found the highest mortality rate in the 25–34 age group [22]. A systematic review corroborated the increased mortality risk in the over-60 age group [OR = 2.57, CI 95% 1.2–5.4] [26]. These findings highlight the vulnerability of older adults in traffic scenarios and suggest the need for age-specific safety measures and urban planning considerations.

The 17–24 age group ranked second in mortality rates, mirroring trends observed in Argentina, Brazil, Chile, and Poland, likely due to riskier behaviors [20, 27–30]. This consistent pattern across multiple countries underscores the importance of targeted interventions for young adults, such as improved driver education and awareness campaigns about the dangers of risky driving behaviors.

The COVID-19 pandemic 2020 significantly impacted TA mortality trends, with a substantial decrease across all provinces and age groups (a variation of 4.1 points compared to 2019). The over-60 age group experienced the most significant reduction, from 30.8 per 100,000 inhabitants in 2019 to 19.9 per 100,000 in 2020 [35]. This trend aligns with findings from Peru, where TA mortality experienced the most significant decline among external causes of death, particularly during the initial 40 days of confinement [36]. These findings suggest that lockdown measures significantly protected traffic-related mortality, particularly for vulnerable age groups.

### Mortality by type of accident

A significant limitation in our study was the high proportion (54.9%) of TA mortalities classified as “V89 - Accident in another type of unspecified transport”. This underreporting and misclassification of TA types leading to mortality complicates understanding the problem’s magnitude and limits regional data comparison [34, 38]. Despite initiatives by PAHO and the Latin American and Caribbean Network for Strengthening Health Systems (RELACSIS) to train health personnel in proper death certificate completion as per WHO standards, this remains a challenge that needs to be addressed to improve data quality and analysis precision [38].

Pedestrian fatalities emerged as the second leading cause of mortality in our study, with a TAMR of 3.44 per 100,000 inhabitants, exhibiting a decreasing trend year-over-year. This aligns with findings from another study in Ecuador, Brazil, and Chile, where pedestrians were the most frequent victims among vulnerable road users [13, 39, 40]. However, this trend contrasts with data from Cali, Colombia, where an increase in mortality was observed [41]. The vulnerability of older adults as pedestrians was particularly notable, with a TAMR of 11.21 per 100,000 inhabitants for those ≥ 60 years old, also demonstrating a decreasing trend [42]. These findings collectively emphasize the importance of continued focus on pedestrian safety in urban planning and traffic safety policies across Latin America, especially for older adults.

Our study highlights the significant mortality burden associated with motorcycle-related TA; motorcyclists represented the third leading cause of TA-related deaths, with an increasing trend and a TAMR of 2.72 per 100,000 persons. This upward trend may be attributed to the rising number of motorcyclists in our country in recent years, particularly among delivery riders, who often engage in high-risk road behaviors [43]. Interestingly, our findings diverge from a study conducted in São Paulo, which reported a stationary mortality trend for motorcyclists [44]. This contrast underscores the variability in urban traffic dynamics and the potential influence of local factors on mortality patterns.

A broader perspective is provided by a study encompassing the Americas from 1998 to 2010, which reported an AMR of 1.6 per 100,000 inhabitants for motorcycle-related fatalities. This rate is notably lower than our findings, suggesting a potential worsening of the situation in our study area over time. The Americas study observed an overall increasing trend in motorcyclist mortality across the entire region, particularly pronounced in Andean countries, with Ecuador leading the group, demonstrating a staggering 78.3% increase [45]. This alarming trend highlights the urgent need for targeted interventions to improve motorcycle safety in Ecuador and the broader Andean region.

Cyclist fatalities, though ranking sixth in our study, warrant careful consideration. We observed a TAMR of 0.21 per 100,000 inhabitants, with a slight upward trend, though not statistically significant. These findings align with a previous study in Ecuador between 2004 and 2017 [46]. In contrast, a study in Colombia revealed a more alarming trend, documenting a 24% increase in cycling-related traffic fatalities from 1998 to 2019 [47]. While the absolute numbers may appear relatively low compared to other traffic-related fatalities, these deaths represent a significant mortality burden that should not be overlooked. The gradual increase may indicate emerging trends in cycling adoption or changes in urban traffic patterns that warrant further investigation and proactive safety measures.

### Inequality analysis

Our analysis revealed lower literacy rates and per capita income are associated with higher TA mortality risks. This finding echoes results from studies in Quito [48], Canada [49], Norway [50], and the United States [51], underscoring the complex interplay between socioeconomic factors and TA mortality. A study in Quito highlighted that a small proportion of the deceased (0.49%) belonged to a high socioeconomic status, while the majority (76%) belonged to a lower socioeconomic status [48].

An exploratory literature review revealed significant disparities in road traffic mortality rates associated with educational attainment and economic status. The findings indicate that populations with lower levels of academic achievement experience disproportionately higher rates of traffic-related fatalities compared to their more educated counterparts. Similarly, a marked inequality was observed when examining mortality rates through the lens of economic stratification, with lower-income populations suffering from elevated mortality rates due to road traffic incidents relative to more economically advantaged demographic groups [52].

These results underscore a critical socioeconomic gradient in TA-related mortality, suggesting that both educational and economic factors play substantial roles in determining vulnerability to fatal road accidents. This pattern of inequality highlights the complex interplay between social determinants of health and traffic safety outcomes. Such findings have important implications for public health policy and road safety initiatives, emphasizing the need for targeted interventions that address these socioeconomic disparities.

While factors such as speed reduction, use of seat belts, child seats, helmets for motorcyclists, and refraining from driving under alcohol and drugs are mandatory in Ecuador [6, 33], adherence to these measures likely varies across demographic groups. This variability represents a limitation in our study, as we did not comprehensively assess these factors. Future research should analyze these variables and their influence on TA mortality in the Ecuadorian population, particularly given that driving under the influence of alcohol significantly increases the risk of TA, as observed in several Latin American countries [53].

### Limitations

Considering the thorough examination conducted in this study, it is essential to acknowledge the potential vulnerability to ecological fallacy, an inherent risk when interpreting data aggregated at the group level, which may not fully capture individual-level nuances. However, it is crucial to emphasize that despite this inherent limitation, the integrity and robustness of the research findings remain steady and unaffected. The methodological rigor employed, alongside the data analysis techniques, ensures that the conclusions drawn contribute to understanding the subject matter. Thus, while acknowledging this potential limitation, the study’s outcomes are valuable insights into the prevailing trends and patterns, bolstering the scientific discourse on the topic.

The limitations encompass various aspects, including the reliance on secondary data sources to ascertain both mortality rates stemming from traffic-related injuries and the multifaceted elements associated with helmet and seat belt usage, driving under the influence of alcohol and drugs, and speed regulation, which warrants a more thorough examination. Moreover, the incomplete data regarding the vehicle fleet’s age distribution and the state and safety features of vehicles and motorcycles present notable hurdles. Additionally, the lack of comprehensive information concerning the equipment available in ambulances for patient care and inadequate insights into the condition of Ecuador’s road infrastructure underscores the necessity for more extensive scrutiny. Mitigating these restrictions is fundamental for propelling the ongoing research endeavors within this domain forward.

## Conclusions

This study is one of the few conducted in Ecuador that aimed to describe and analyze the trends in mortality due to TA. It has found an annual decrease in mortality rates during the study period, with a more significant decline among men in the Amazon and Coast regions and the age group of 60 years and older. There is evidence of substantial underreporting of the causes of death. The pedestrian group is the most affected after excluding the leading cause (V89 - Accident in another type of unspecified transport). However, there has been a decrease in recent years, where motorcyclists exhibit higher mortality despite reforms to traffic laws made over these ten years.

Furthermore, this is the first study on inequalities in TA in Ecuador, in which we have conducted an analysis combining descriptive, associative, and inequality measures. We hope to provide the necessary information for decision-makers to prioritize this public health issue.

An exciting finding relates to vulnerable road users over 60 years of age, who show the highest mortality rates. Although the overall rate in this group tends to decrease over the entire 12-year period, their vulnerability deserves special attention.

There are socioeconomic inequalities in mortality rates, but it is necessary to study them more deeply to achieve the desired impact on reducing mortality due to traffic accidents. From these data, public policies could be generated.

## Data Availability

The data that are presented in this study are available on request from the corresponding author. The data are not publicly available due to maintaining privacy data of the participants such as e-mail addresses.

## Abbreviations

AG: Absolute gaps
AR: Absolute risks
APV: Annual percentage variation
ICD-10: 10th revision of the International statistical classification of diseases and related health problems
GDP: Gross domestic product
RELACSIS: Latin american and caribbean network for strengthening health systems
INEC: National institute of statistics and census of the republic of Ecuador
PCI: Per capita income
RG: Relative gaps
SII: Slope inequality index
SDGs: Sustainable development goals
TAMR: Traffic accident mortality rates
TA: Traffic accidents
WHO: World health organization
